# The Acceptability of Technology in Health Care Among Older Korean Adults With Multiple Chronic Conditions

**DOI:** 10.1093/geroni/igaa057.720

**Published:** 2020-12-16

**Authors:** Ji Yeon Ha, Hyeyoung Park

**Affiliations:** 1 KONYANG UNIVERSITY, Daejeon, Republic of Korea; 2 University of Massachusetts Amherst, Amherst, Massachusetts, United States

## Abstract

Background: Although there are benefits in utilizing ICT in health care, older adults have challenges in employ technologies in their health care management due to the changes in cognitive and physical functions, low motivation to use technology, and low computer/internet literacy (Adebayo et al, 2017; Wildenbos et al, 2018). The purpose of this study is to investigate the acceptance of technology among older Korean adults with multiple chronic conditions and examine factors associating with the acceptance of the technology. Method: The participants were 226 community-dwelling older adults who have more than two chronic conditions. Directed by the senior technology acceptance model (Chen & Chan, 2014), demographics, gerontechnology self-efficacy, gerontechnology anxiety, facilitating conditions, self-reported health conditions, cognitive ability, social relationship, attitude to life and satisfaction, physical functioning, and acceptance of technology were surveyed using a self-reported questionnaire. Findings: Older Korean adults with multiple chronic conditions showed a moderately high technology acceptance score (M = 25.35, SD = 5.28). There were significant differences in the acceptance of technology depending on age (r=-0.241, p<.01), cognitive ability (r=0.225, p<.01), gerontechnology self-efficacy (r=0.323, p<.0001), and facilitating conditions (r=0.288, p<.0001). Conclusion: While older age were associated to the acceptance of technology, gerontechnology self-efficacy which is one’s judgment of their ability to perform a task successfully using gerontechnology and facilitating conditions which are environmental factors that help older adults use gerontechnology easier were positively associated with the acceptance of technology among older Korean adults with multiple chronic conditions.

